# New Insights into the Epidemiological Characteristics of *Mycoplasma pneumoniae* Infection before and after the COVID-19 Pandemic

**DOI:** 10.3390/microorganisms12102019

**Published:** 2024-10-05

**Authors:** Qianyue Wu, Xiaozhou Pan, Dingding Han, Zhan Ma, Hong Zhang

**Affiliations:** 1 Clinical Lab in Children’s Hospital of Shanghai, Children’s Hospital of Shanghai Jiao Tong University, Shanghai 200040, China; wuqianyue@sjtu.edu.cn (Q.W.); sherry_pxz@sjtu.edu.cn (X.P.); handingding@shchildren.com.cn (D.H.); maz@shchildren.com.cn (Z.M.); 2 Institute of Pediatric Infection, Immunity, and Critical Care Medicine, Shanghai Jiao Tong University School of Medicine, Shanghai 200062, China

**Keywords:** *Mycoplasma pneumoniae*, epidemiological, genotypes, macrolide resistance

## Abstract

*Mycoplasma pneumoniae* (*M. pneumoniae*), a prevalent respiratory pathogen affecting children and adolescents, is known to trigger periodic global epidemics. The most recent significant outbreak commenced in the first half of 2023 and reached its peak globally during the autumn and winter months. Considering the worldwide repercussions of the COVID-19 pandemic, it has become increasingly essential to delve into the epidemiological characteristics of *M. pneumoniae* both before and after the pandemic. This review aims to provide a comprehensive analysis of the key features of *M. pneumoniae* epidemics in the pre-and post-COVID-19 contexts, including but not limited to shifts in the susceptible population, the molecular genotypes of the pathogen, the clinical manifestations, and potential new trends in drug resistance. Additionally, we will introduce the latest advancements in the diagnosis of *M. pneumoniae*.

## 1. Introduction

Mycoplasmas are microorganisms that occupy a niche between bacteria and viruses, representing the smallest self-replicating organisms capable of existing without cells. Some of these organisms are pathogenic to humans and animals. *Mycoplasma pneumoniae* (*M. pneumoniae*) is a common pathogen responsible for atypical pneumonia, particularly in school-aged children and adolescents [1]. The detection rate of *M. pneumoniae* in children with community-acquired pneumonia (CAP) ranges from 4% to 39%, as determined by polymerase chain reaction (PCR) or serological methods [2]. *M. pneumoniae* primarily adheres to respiratory epithelial cells through a specialized polarized terminal attachment organelle and induces pathogenic effects by releasing superoxide radicals and hydrogen peroxide [3]. Although *M. pneumoniae* pneumonia (MPP) is generally considered a self-limiting disease, some individuals may experience severe clinical manifestations, referred to as severe MPP (SMPP) [4]. The severity of the disease is associated with immune dysregulation and resistance to macrolides (MRMP). Macrolide resistance is characterized by mutations in the 23S rRNA of *M. pneumoniae*, which reduce the affinity between macrolides and the 50S bacterial ribosomal subunit [5]. The most common mutation identified is A2063G [6]. Notably, there is a geographical disparity in the drug resistance of *M. pneumoniae*, with Asia reporting substantially higher resistance rates compared to Europe and America [7].

*M. pneumoniae* can be classified into various genotypes using a range of molecular typing methods, including P1 typing, multi-locus variable-number tandem-repeat analysis (MLVA), multi-locus sequence typing (MLST), and single nucleotide polymorphism typing (SNP), which is based on whole-genome sequencing technology [8,9]. All genotypes cocirculate during *M. pneumoniae* epidemics; however, the dominant genotype may change alternately every few years, influenced by geographical factors and the varying levels of population immunity to specific genotypes [10,11]. Previous studies have also indicated that different genotypes exhibit varying sensitivity to macrolides [12]. Comparing the molecular genotypes of samples across different periods and geographic locations aids in elucidating the evolutionary relationships of *M. pneumoniae* and enhances our understanding of the dissemination of macrolide-resistant strains.

*M. pneumoniae* infections exhibit an epidemic cycle that occurs every 3–5 years, with a duration of 1–2 years [13,14]. The epidemic cycle may be related to the duration of herd immunity, which lasts about four years before people are again susceptible to infection with *M. pneumoniae* [15]. The prolonged duration of *M. pneumoniae* epidemics may be associated with the pathogen’s long incubation period, relatively low transmission rate, and the persistence of the organism in the respiratory tract [16]. During the COVID-19 pandemic, the prevention and control measures, or non-pharmaceutical interventions (NPIs), blocked the transmission of many respiratory pathogens, including *M. pneumoniae* [17]. Therefore, during the period of NPIs, the detection rate of *M. pneumoniae* was extremely low, until the first half of 2023, when there was an observable resurgence, indicating a new outbreak of *M. pneumoniae* [18].

Considering the COVID-19 pandemic has altered the epidemic patterns of various respiratory pathogens, this article provides a comprehensive review of the epidemic characteristics, clinical manifestations, molecular types, and macrolide resistance of *M. pneumoniae* before and after the COVID-19 pandemic, and of new developments in diagnostics during the period of endemicity.

## 2. Epidemiology of *Mycoplasma pneumoniae* Infections

Research has indicated that the increasing coverage of pneumococcal conjugate vaccines (PCVs) and H influenzae type b (Hib) vaccination has attributed to the largest decline in global infection rates of these pathogens [19]. However, there are no commercially available vaccines for *M. pneumoniae*, which is likely to lead to a relatively increased infection rate, making it a prominent respiratory pathogen in children.

*M. pneumoniae* is an atypical respiratory pathogen with a global cycle of 3–7 years. It had caused outbreaks in 2011–2012, 2014–2015, and 2015–2016 across Asia and Europe [20,21,22]. During periods of endemicity, it may be responsible for 20–40% of CAP cases in the general population, with an increasing prevalence of up to 70% in close populations [1,23]. Before the COVID-19 pandemic, studies had observed an increase in the incidence of *M. pneumoniae* infections from late 2019 to early 2020, indicating a new round of outbreak [18,24,25]. However, NPIs during the COVID-19 pandemic reduced *M. pneumoniae* detection rates [17], with low incidence rates of 1.69%, 0.9%, and 0.82% for 2020, 2021, and 2022, respectively [18,26,27]. The subsequent surveillance dataset revealed a surprising increase in the average rate, which had risen to 4.12% from April to September 2023 [18]. Subsequently, the WHO has noted increased consultations and hospitalizations for MPP in China since May 2023 [28,29]. In late 2023, several European countries reported *M. pneumoniae* outbreaks, drawing global concern [18,30,31].

Specifically, data from Beijing showed that the positive rates reached 25.4% for outpatients, 48.4% for inpatients, and peaked at 61.1% for respiratory patients [32]. In other cities like Wuhan and Guangzhou, China, detection rates were extraordinarily high, at 90% and 60%, respectively [29,33]. These ratios were much higher than the 30% positivity rate in Beijing in 2019 [34]. In contrast, Western countries reported lower rates, with Marseille, France, documenting a positive rate of 0.8% in 2023–2024, mirroring the rate of 0.89% observed in the United States since September 2023 [35,36]. In general, the COVID-19 pandemic has significantly diminished the populace’s immunity to *M. pneumoniae*, potentially facilitating its resurgence and subsequent epidemic state following the relaxation of NPIs.

## 3. Susceptible Population of *Mycoplasma pneumoniae*

The recent outbreaks of *M. pneumoniae* still primarily affect school-aged children aged over five years, who account for nearly 60% of the hospitalized cases of MPP [33,37]. A recent multicenter study on children conducted in South Korea suggests that from September to December 2023, children aged 5–9 years constituted 57.5% of MPP cases [38]. Nevertheless, data from China indicate a significant rise in infections among children under 3 years during the 2023 outbreak, suggesting a concerning shift towards younger demographics being affected [32]. The data show that during the *M. pneumoniae* epidemic in 2023, children aged 0–3 years accounted for 18–30% of MPP cases [33,35,37]. This increase may largely be attributed to NPIs that have left younger children largely unexposed to *M. pneumoniae*, resulting in a lack of protective antibodies and increased susceptibility to the pathogen. Furthermore, during this resurgence, the extensive application of PCR technology has significantly decreased the previous rate of missed diagnoses, particularly among younger children, whose immunological response is inadequate to generate sufficient antibody levels.

Moreover, children may act as asymptomatic carriers, potentially leading to small outbreaks within familial settings. Historical data from the United States indicate that the proportion of adults in *M. pneumoniae* infections varies from approximately 0.5% to 2% [39,40]. However, in 2023, it was observed that 19%(131/683) of adults aged 19–39 years with confirmed *M. pneumoniae* infections required hospitalization in Denmark [41]. Similarly, Marseille, France, has reported an increase in MPP cases among adults since January 2024 [35].

## 4. Molecular Genotypes and Macrolide Resistance of *Mycoplasma pneumoniae*

According to the sequence variations in the P1 adhesin gene (MPN140 to MPN142), *M. pneumoniae* can be classified into two genotypes: P1-1 and P1-2. The distribution of genotypes in a population varies due to geographical environmental factors [10]. For instance, in Japan, there is an approximate transition of P1 type every decade [42]. Meanwhile, in China, P1-1 has remained the most frequently identified genotype both before and after COVID-19, accounting for 80–90% of cases pre-pandemic [43,44] and 70–90% post-pandemic [45,46]. Nonetheless, some studies reported P1-2 as dominant, even stating that it accounted for 74% of cases post-COVID-19 pandemic [23,47]. It is speculated that the lack of evident genotype conversion in China may stem from a deficiency in comprehensive, large-scale epidemiological data. Currently, molecular typing studies in other countries are scarce, highlighting a need for more extensive global research. 

Since MRMP was first identified in Japan in the early 2000s, its prevalence has escalated significantly, rising from 18.2% in 2000 to 41.0% in 2010, and further surging to 76.5% by 2019 [7]. In Asia, particularly across most regions of China, the incidence of MRMP is alarmingly high, exceeding 90%, posing a considerable challenge to clinical practices [48]. In Europe and the United States, the detection rate of MRMP ranges from 1% to 30% from one country to another [40]. After the COVID-19 pandemic, the prevalence of MRMP in China remains high [34]. Meanwhile, in the United States and some European countries like Denmark, the detection rates of MRMP remain low, recorded as 7.14% and 1.5%, respectively [36,41]. 

Research indicates a correlation between the prevalence of MRMP and the prescription rates of macrolides, as well as different genotypes [49]. Before the COVID-19 pandemic in China, the P1-1 genotype demonstrated a substantial macrolide resistance rate of approximately 90%. In contrast, MRMP prevalence of the P1-2 genotype experienced a significant increase from 20% in 2015 to 93.48% by 2019 [10,44]. After COVID-19, the resistance rate of P1-1 persisted at a high level of 90–100%, while the drug resistance of P1-2 continued to climb, surpassing 95% [45,47]. A recent phylogenetic analysis by Li et al. identified two predominant epidemic clones in China: the EC1 clone associated with P1-1 and the EC2 clone linked to P1-2 [50]. Notably, the EC2 clone exhibited complete macrolide resistance [50]. Moreover, EC2 may be a drug-resistant strain derived from the P1-2 type under the pressure of macrolide antibiotics, and its widespread proliferation may account for the prevalent resistance observed in the P1-2 genotype. The pronounced macrolide resistance among *M. pneumoniae* strains presents significant challenges in China, especially for the pediatric population, for whom effective treatment options are limited.

## 5. Clinical Presentations of *Mycoplasma pneumoniae* in the COVID-19 Context

*M. pneumoniae* infection can affect both the upper and lower respiratory tracts, causing relevant clinical manifestations including fever, sore throat, paroxysmal dry cough, and headache. These symptoms are remarkably like those of severe acute respiratory syndrome coronavirus 2 (SARS-CoV-2) infection [51].Therefore, differential diagnosis is difficult based only on clinical presentations; not to mention, there are also SARS-CoV-2–*M. pneumoniae* co-infection cases [51]. In Gayam’s study, all six patients with co-infection exhibited fever, and nearly all displayed symptoms of cough, shortness of breath, and fatigue; all inflammatory markers were elevated, and the chest X-ray at presentation showed bilateral infiltrates in all the patients (100%) [52]. Nonetheless, during the COVID-19 pandemic, the prevalence of SARS-CoV-2–*M. pneumoniae* co-infection was relatively low and showed variation; for instance, in Qingdao and Wuhan, China, the rates were 23.3% and 2.63% [53], respectively, while in the United States, Spain, and the United Kingdom, they were 1.7%, 0.97%, and 1.49%. Additionally, this ratio appears to be as high as 16/34 (47.0%) within the pediatric population [54].

Research indicates that patients with bacterial co-infections had poorer respiratory conditions compared to those with isolated SARS-CoV-2 infections, and more intensive care unit admissions [55]. Meanwhile, Zha et al. found through comparative research that there were no significant associations between *M. pneumoniae* co-infection and major clinical manifestation on admission [56]. However, the risk of thrombosis was significantly increased by the co-infection with *M. pneumoniae* [56]. This latter finding has raised our concerns significantly.

In fact, *M. pneumoniae* infection may induce a systemic hypercoagulable abnormality. The specific mechanisms may include inflammation, which damages the endothelial cells and activates the coagulation system, immune-mediated injury, antiphospholipid syndrome, and liver damage [57]. Laboratory tests indicate that co-infection results in elevated levels of D-dimer and fibrinogen, consequently increasing the risk of thrombosis compared to in patients infected solely with COVID-19 [55]. This hypercoagulability may influence the degree of pulmonary infiltration to some extent. Li et al. indicated through retrospective studies that fibrinogen and D-dimer levels changed most significantly in the lung consolidation group [57], and Luo et al. reported that D-dimer was associated with lung necrosis, as well as that patients with elevated D-dimer were significantly more likely to have longer times for radiographic clearance [58]. In certain patients with severe hypercoagulability, pulmonary embolism may occur, posing a life-threatening risk [59]. Therefore, it is clinically essential to integrate real-time laboratory coagulation tests to determine the necessity of anticoagulant therapy.

## 6. The Prevalence of Severe *Mycoplasma pneumoniae* Pneumonia 

The diagnosis of SMPP focuses on the severity of the disease, and patients with SMPP usually suffer more severe intrapulmonary or extrapulmonary symptoms and elevated inflammatory indicators such as C-reactive protein (CRP), lactate dehydrogenase (LDH), and D-dimer compared to those with general MPP (GMPP) [60]. In recent years, the incidence of SMPP has increased, and this has primarily been attributed to the proliferation of macrolide-resistant strains, especially among pediatric populations [61,62]. 

Before the COVID-19 pandemic, the occurrence of SMPP was generally over 10% [37,63,64], though there was an exception in that a Beijing epidemiological study reported 42.6% of MPP cases as SMPP in 2016 [63]. During and after the COVID-19 pandemic, there was an increase in the prevalence of SMPP during the resurgence of *M. pneumoniae* infections, which significantly burdened healthcare facilities. Several studies have reported that the prevalence of SMPP surpassed 20% [37,47], and alarmingly, in a hospital in northern China, even hit 50% [61].

It is speculated that the increased proportion of SMPP during the resurgence of *M. pneumoniae* may be linked to the immunological impairment caused by COVID-19 infection [37]. Research indicates that after infection with the novel coronavirus, there is a prolonged phase of immune suppression and damage from inflammatory response, manifesting in a reduction in the counts of natural killer (NK) cells, lymphocytes, and monocytes, along with increased inflammatory cytokines [65,66]. Notably, research points to immune dysregulation and inflammatory damage as the main pathogenic mechanisms of SMPP [37,61]. This suggests that immunological impairment induced by COVID-19 may escalate the risk of patients with MPP progressing to SMPP. The precise mechanisms involved warrant further exploration.

SMPP may also be observed in other patient populations, including both the elderly and young adults without any underlying diseases [67,68]. Indeed, during the post-COVID-19 period, there has been a reported surge in SMPP cases among adults in Europe, including individuals in their 30s and 40s who previously had no chronic health conditions. These patients exhibited acute symptoms, including fever, dyspnea, cough, and pulmonary infiltrates. As their condition deteriorated, many patients were susceptible to respiratory failure, necessitating mechanical ventilation for stabilization [69,70,71].

## 7. Diagnosis

The diagnosis of *M. pneumoniae* requires consideration of multiple factors, including clinical manifestations, laboratory tests, and radiological examination. At present, clinical laboratories frequently employ serological assays and nucleic acid amplification tests (NAATs) for the detection and diagnosis of *M. pneumoniae* infection. For more data, refer to Table 1.

Serological methods diagnose *M. pneumoniae* by monitoring the changes in IgM or IgG titers during the acute and convalescent phases of the disease [77,78]. However, serological methods may produce false negatives and false positives, particularly in immunocompromised patients or when specimens are obtained too early [1,79]. 

NAAT mainly focuses on the amplification of adherent gene P1 alongside additional genetic targets, such as community-acquired respiratory distress syndrome toxin (CARDS) genes and noncoding repetitive elements of the pathogen [1]. Specimens for NAAT can be obtained from both the upper and lower respiratory tracts, including but not limited to nasopharyngeal swabs, sputum, and bronchoalveolar lavage fluid [80]. NAAT is extensively employed in clinical laboratories due to its shortened turnaround time, enhanced sensitivity and specificity, and capacity to yield quantitative results [1]. Particularly in the early phase of infection when specific antibodies are yet to be produced, nucleic acid testing delivers more precise results. Conversely, in the later stages of infection, the residual DNA from dead *M. pneumoniae* may lead to false-positive outcomes [80]. Moreover, the limitations of NAAT include high detection costs, stringent testing prerequisites, and interference from multiple primers [78,81,82]. 

Driven by the COVID-19 pandemic, molecular detection technologies have progressed rapidly, exemplified by next-generation sequencing (NGS) in diagnosing various respiratory pathogens. For primary healthcare hospitals, innovative patterns of NAAT, such as loop-mediated isothermal amplification (LAMP) and CRISPR, are progressing towards point-of-care testing (POCT) [83,84]. For instance, LAMP technology operates without the need for thermal cycling, enabling nucleic amplification under isothermal conditions of 60–65 °C. The entire testing process takes only 15 to 60 min and does not require complicated equipment [85,86]. Similarly, some CRISPR-based diagnostics not only possess excellent pathogen-specific recognition capabilities but also offer intuitive visualization of results through colorimetric changes or immunochromatographic test strips [87,88].

In order to distinguish the acute, chronic, and carrier states of *M. pneumoniae* infection [89], researchers have developed a method for the detection of antibody-secreting cells (ASCs) in peripheral blood mononuclear cells for the diagnosis of MPP [90]. The ASCs emerge two days after the onset of infection symptoms and decrease in number as recovery progresses [91,92,93]. The method of detecting specific ASCs through the ELISpot technology exhibits a sensitivity of 90% and a specificity of 80% [90]. However, the method is hampered by a complex sample processing procedure and an incomplete understanding of the timing of ASCs’ response [94]. Nonetheless, the ASCs ELISpot assay, as an emerging diagnostic biomarker, holds great potential in the future.

## 8. Conclusions

In summary, *M. pneumoniae* is one of the most common pathogens causing CAP, particularly prevalent among children and adolescents. The COVID-19 pandemic has compromised immunity levels within the community, resulting in heightened vulnerability to *M. pneumoniae* infections. The autumn and winter of 2023 witnessed a worldwide epidemic of *M. pneumoniae*, characterized by a noticeable shift towards younger age groups and a high incidence of MRMP and SMPP, particularly in Chinese regions. In the future, it is essential to deepen clinical understanding of *M. pneumoniae* infection and to keep a close watch on its epidemiological trends. Additionally, prompt diagnosis and the exploration of alternative treatment strategies will emerge as pivotal clinical priorities.

## Figures and Tables

**Table 1:** Literature data on *M. pneumoniae* infection during and post-COVID-19.

Author	Year of Publication	Clinical Presentations	Radiological Finding	Diagnostic Method	Increased Laboratory Markers	Outcome
Fan et al. [72]	2020	NA	NA	Serology	Lymphopenia **, Thrombocytopenia **,	ICU *
Chen et al. [73]	2020	Fever, cough	Infiltrate, pleural effusion	Serology	D-dimer	NA
Gao et al. [74]	2020	Cough, debilitation	Bilateral infiltrate	Serology	CRP, ESR *	Recovery
Gayam et al. [52]	2020	Fever, cough, shortness of breath, fatigue	Bilateral infiltrates	Serology	Lymphopenia **, ESR, CRP, D-dimer	ICU *, recovery
Garzoni et al. [71]	2024	Fever, dyspnea	NA	qPCR	CRP	Recovery
Jiang et al. [47]	2023	Fever, cough, dyspnea	Consolidation, pleural effusion	qPCR	CRP, LDH, D-dimer	NA
Larcher et al. [70]	2024	Fever, dyspnea, cough	Infiltrates, consolidation	qPCR	NA	ICU *, recovery
Zhang et al. [37]	2024	Fever, pleural effusion	NA	Serology qPCR	CRP, LDH, D-dimer	NA
Zayet et al. [75]	2024	Fever, dyspnea, cough,	Bilateral consolidation	serology qPCR	CRP	Recovery
Lee et al. [38]	2024	High fever, dyspnea	Consolidation, pleural effusion	Serology qPCR	CRP, ESR *, LDH	Recovery
Kyi et al. [33]	2024	Fever, cough	Consolidation/Atelectasis	tNGS *	CRP	NA
Xing et al. [76]	2024	Fever, cough	Boundaries or interstitial changes	qPCR	CRP	Recovery

* ESR (erythrocyte sedimentation rate); tNGS (targeted next-generation sequencing); ICU (intensive care unit). ** Lymphopenia/thrombocytopenia, the reduction in lymphocyte count and platelet count; these two articles regard SARS-CoV-2–*M. pneumoniae* co-infection.

## Data Availability

No new data were created or analyzed in this study.
